# Effectiveness of alcohol brief intervention delivered by community pharmacists: study protocol of a two-arm randomised controlled trial

**DOI:** 10.1186/1471-2458-13-152

**Published:** 2013-02-18

**Authors:** Ranjita Dhital, Ian Norman, Cate Whittlesea, Jim McCambridge

**Affiliations:** 1 King’s College London, Florence Nightingale School of Nursing and Midwifery, 57 Waterloo Road, SE1 8WA, London, UK; 2 King's College London, King’s Health Partners, Pharmaceutical Science Clinical Academic Group, Institute of Pharmaceutical Science, Franklin-Wilkins Building, 150 Stamford Street, SE1 9NH, London, UK; 3 London School of Hygiene & Tropical Medicine, Department of Social and Environmental Health Research, Room 237, 15-17 Tavistock Place, WC1H 9SH, London, UK

**Keywords:** Alcohol, Brief intervention, Community pharmacist, Community pharmacy, Hazardous and harmful drinking

## Abstract

**Background:**

There is strong evidence to support the effectiveness of Brief Intervention (BI) in reducing alcohol consumption in primary healthcare.

**Methods and design:**

This study is a two-arm randomised controlled trial to determine the effectiveness of BI delivered by community pharmacists in their pharmacies. Eligible and consenting participants (aged 18 years or older) will be randomised in equal numbers to either a BI delivered by 17 community pharmacists or a non-intervention control condition. The intervention will be a brief motivational discussion to support a reduction in alcohol consumption and will take approximately 10 minutes to deliver. Participants randomised to the control arm will be given an alcohol information leaflet with no opportunity for discussion. Study pharmacists will be volunteers who respond to an invitation to participate, sent to all community pharmacists in the London borough of Hammersmith and Fulham. Participating pharmacists will receive 7 hours training on trial procedures and the delivery of BI. Pharmacy support staff will also receive training (4 hours) on how to approach and inform pharmacy customers about the study, with formal trial recruitment undertaken by the pharmacist in a consultation room. At three month follow up, alcohol consumption and related problems will be assessed with the Alcohol Use Disorders Identification Test (AUDIT) administered by telephone.

**Discussion:**

The UK Department of Health’s stated aim is to involve community pharmacists in the delivery of BI to reduce alcohol harms. This will be the first RCT study to assess the effectiveness of BI delivered by community pharmacists. Given this policy context, it is pragmatic in design.

**Trial registration:**

Current Controlled Trials ISRCTN95216873

## Background

In the UK alcohol misuse leads to an estimated cost to society of £25.1 billion per annum (NHS costs £2.7 billion) and is the third leading cause of ill health [1]. Population-level interventions that seek to influence the price, availability and cultural acceptability of heavy drinking are likely to be most effective in reducing these problems [2]. These may be complemented by individual-level interventions delivered in health services and elsewhere. The UK Department of Health aims to involve community pharmacists in delivering alcohol brief interventions [3]. It has recommended that pharmacy based alcohol interventions should be piloted and evaluated.

Brief interventions are discussions which seek to change views of the personal acceptability of excessive drinking and to encourage self-directed behaviour change. They include simple forms of structured advice and brief counselling. Typically, questions about alcohol use are asked to motivate the person to take action to change drinking where this may be beneficial [4]. There is strong evidence to support the effectiveness of BI to reduce alcohol consumption in primary healthcare [5]. In 21 randomised controlled trials conducted in primary health care settings with 7,286 participants, those who received BI reduced their alcohol consumption by 41 grams/week (five U.K. units of alcohol) on average compared to those who did not receive BI [5]. However there are no trials which have assessed the effectiveness of BI delivered in the community pharmacy setting.

It is useful to think of three types of drinking that are injurious to health: a) Hazardous drinking (which carries a risk of harmful consequences to the drinker for example occasional binge drinking); b) Harmful drinking (pattern of drinking already causing psychological or physical damage to health) and c) Dependent drinking (which may benefit from specialist intervention) [1]. The World Health Organisation (W.H.O) 10-item Alcohol Use Disorder Identification Test (AUDIT) screening tool has been extensively validated in identifying those whose drinking is hazardous or harmful, including those who are dependent [6-8].

Guided by the Medical Research Council (MRC) framework for developing and evaluating complex interventions [9], the design of this trial has been informed by our previous studies assessing pharmacy customers’ perceptions and the feasibility of BI in community pharmacies [10,11] and studies that recommend establishing the acceptability of discussing alcohol use in health care settings [12]. Our NHS Westminster study which assessed 102 pharmacy customers perceptions of BI established that most customers (*N* = 97, 96%) would find it acceptable to discuss their drinking with the pharmacist [10]. Our uncontrolled before and after study of BI in community pharmacies in Lambeth involved training 29 community pharmacists in BI and monitoring change in drinking among a cohort of service users [11]. Experiences of receiving the service, and the barriers and enablers experienced by study pharmacists were also assessed. A key finding of this study was that pharmacists unfamiliar with BI could be trained to deliver alcohol interventions. In addition involving support staff to inform pharmacy customers about BI and providing regular support to pharmacy staff were found to be important factors in achieving high BI delivery rates in community pharmacies.

This trial builds on previous research undertaken by others, including a questionnaire study in New Zealand exploring attitudes, knowledge and experiences of 101 community pharmacists. These pharmacists reported being motivated to take up BI but expressed a lack of confidence, knowledge and skills to advise customers on their drinking [13]. A larger study conducted in New Zealand (2383 customers at 43 pharmacies), also suggested that customers were positive about being offered BI from pharmacies [14].

There has been one published discussion paper assessing the feasibility of BI conducted by community pharmacists in the UK [15]. The three feasibility studies discussed in this review (all U.K. cities: London; Glasgow; and Leeds) included a total of 14 pharmacies and 500 customers, from which 30% to 53% were identified as drinking above the UK recommended levels (i.e. women drinking more than 24 grams of ethanol/day and men more than 32 grams of ethanol/day [1]. The authors in the discussion paper supported the feasibility of BI delivered in community pharmacies and noted that there had been “little empirical evaluation of the effectiveness of community pharmacy-based services for alcohol misuse”, concluding that large scale effectiveness studies were now needed [15].

### Aim

To determine if alcohol BI delivered by community pharmacists is effective at reducing hazardous and harmful drinking among pharmacy customers at three-month follow-up compared to a non-intervention leaflet-only control condition.

### Objectives

● To identify the pharmacy use and demographic profiles of participants recruited to the trial and pharmacy customers who did not fulfil study inclusion criteria or who refused.

● To determine rates of recruitment, refusal and retention among pharmacy customers who are approached to enter the trial.

● To conduct a randomised controlled trial of the effectiveness of alcohol BI for hazardous and harmful drinkers accessing community pharmacy services within NHS Hammersmith and Fulham, London, UK.

● To assess differences in risky drinking and general health status between BI and control participants after three-months.

● To determine participants’ experience of participating in this trial.

## Method and design

### Trial design

**Figure 1:**
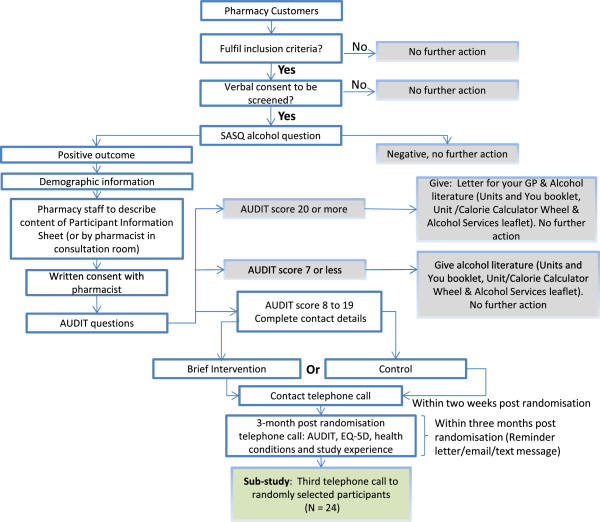
**Study design.** Measures used with trial participants and the stage that they are administered.

This study is a two arm randomised controlled trial (Figure 1). Pharmacy customers who have consented to participate in the trial will have their alcohol risk assessed using the AUDIT [6]. The target population are hazardous and harmful drinkers scoring 8 or over and less than 20 (at which score it is recommended that alcohol dependence be assessed).

### Participants

#### Setting

The London borough of Hammersmith and Fulham has a population of 177100, which comprises a high proportion of young adults (45% in their 20’s and 30’s) and is ethnically diverse [16]. There are 40 community pharmacies within Hammersmith and Fulham [17], of which 37 provide a smoking cessation service, 14 supervised consumption service of controlled medications (e.g. methadone) and 11 provide a needle exchange service [17]. Local needs assessment suggests that 31.5% *(N* = 45914) of Hammersmith and Fulham residents are hazardous or harmful drinkers and have the highest rates of harmful and binge drinking behaviour in London [16].

#### Sample size

Sample size consideration was based on a meta analytic effect size of 0.30 in non-treatment seeking samples at three-month follow-up in the review of brief interventions across settings undertaken by Moyer and colleagues [18]. Using G Power computer programme [19], requiring 80% power in a one-tailed power calculation with alpha = 0.05, 139 participants per group would be required to detect an effect of this magnitude. Allowing for 30% attrition at three months, 199 participants per group will be recruited (a total of 398 trial participants).

**Figure 2:**
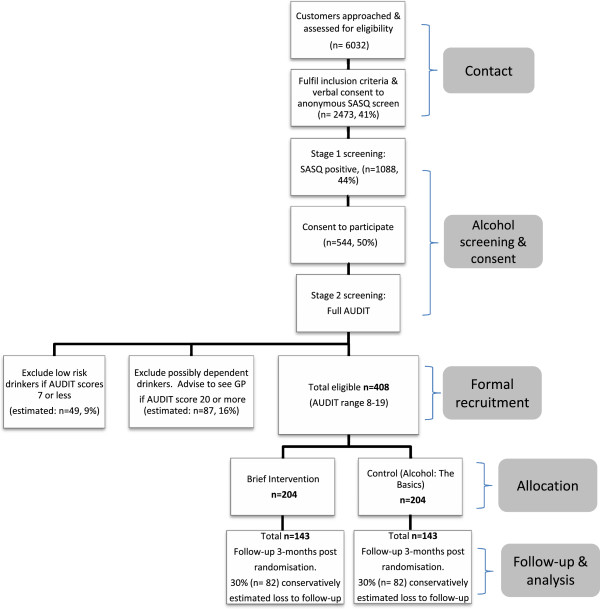
**Study participant recruitment.** Expected number of pharmacy customers to be: approached, assessed for eligibility, recruited to the trial, allocated an intervention or control condition and followed up for analysis.

Findings from a preliminary study identified that 41% (*N* = 98) of pharmacy customers approached to participate in a survey were willing to accept anonymous screening of their alcohol use [10]. We conservatively estimated that approximately 6032 pharmacy customers would need to be approached and invited to participate, for 2473 to agree to be screened. Based on findings from previous feasibility studies [11,15,20] we expect approximately 44% (*N* = 1088) to be identified as risky drinkers with the Single Alcohol Screening Question (SASQ; see below) preliminary screen. We estimate that 50% *(N* = 544*)* of those who screen positive will consent to participate in the study and that 25% (*N* =136) of these will be found not to be eligible when the AUDIT is administered in the second stage of the procedure. Sample size estimates at each stage of the recruitment process for a projected final *N* = 408 are presented in Figure 2.

#### Community pharmacist recruitment

Pharmacists practicing in community pharmacies within Hammersmith and Fulham, with an NHS contract and a private consultation room will be invited to participate. Pharmacists will be contacted initially by letter and telephone. Following this, personal pharmacy visits will be arranged to enable the Chief Investigator (RD) to provide information on the trial, obtain pharmacists’ consent and arrange suitable training dates. A total of 17 pharmacists will be recruited for the study. Pharmacy support staff working with recruited pharmacists will be invited to attend a brief training session on how to inform and identify suitable participants for the trial.

#### Participant recruitment

Pharmacy customers will be informed about the study by pharmacy staff if they are observed performing any of the following activities: a) viewing study posters and flyers displayed within the pharmacy counter area; b) making a general health query or seeking advice which could be linked to alcohol use; c) purchasing pharmacy over the counter products for smoking cessation aids, gastrointestinal remedies, sleep aids and central nervous system depressants (listed in the current edition of the *Medicine Chest* directory [21] d) receiving any of the following pharmacy services: Smoking Cessation, Medication Use Review, Health Check or Emergency Hormonal Contraception; e) presenting prescriptions for medications for any of the following conditions: cardiovascular disease, depression or anxiety, diabetes or gastric problems.

Records of number of customers informed about the study but who refuse to participate or do not fulfil the study criteria will be kept. Anonymous information on customers’ gender, activity in the pharmacy, reason given for not participating and the date they visited the pharmacy will be recorded. Customers will have the content of the Participant Information Sheet explained by pharmacy staff and be given a copy to keep and be asked a Single Alcohol Screening Question (SASQ), “*How often do you have three or more drinks on a single occasion*?” This question was adapted for this trial and is based on the previously validated SASQ [22,23]. We are aware that such brief screeners have their limitations and thus we used a two stage eligibility procedure [24]. Customers who are administered the SASQ and report consuming three or more drinks monthly or more regularly will be invited to have their alcohol use assessed further using the AUDIT by the pharmacist in the consultation room. Participants who fulfil the study criteria and are willing to participate in the trial will sign two copies of the consent form with the pharmacist in the consultation room and will be given one copy to keep.

#### Selection criteria

People aged 18 years or over accessing pharmacy services within the participating pharmacies located in NHS Hammersmith and Fulham who: a) have AUDIT scores between 8–19 inclusive; b) are contactable by phone during the study; c) have a home address in the UK; and d) are able to speak, read and write in English are eligible to take part in the study. Excluded are those who are: a) currently in treatment for alcohol problems; b) currently involved in any other alcohol research; and c) employees of the trial pharmacies.

Pharmacy customers will thus be excluded if they are identified as being possibly alcohol dependent (AUDIT score ≥ 20). This group will be advised to see their GP and be given a letter, with their AUDIT assessment result, to take to their GP. Pharmacy customers who are identified as low risk drinkers (AUDIT ≤ 7) will also be excluded from the study. Both excluded groups will be given, “Units and You” booklet [25], a “Unit/Calorie Calculator Wheel” [26] and an alcohol services leaflet developed for the study to take away.

### Interventions

#### Brief intervention

Participants allocated to the Brief Intervention (BI) condition will be offered a discussion of approximately 10 minutes duration [4]. This intervention contains a number of structured components in the form of an intervention protocol. The conversation begins by building rapport through asking questions about participants’ experience of answering the AUDIT screening questions. Participants are then encouraged to talk about how drinking fits in with their lives, explore any ambivalence and elicit their evaluation of their drinking including any problems associated with it. The conversation will close by either the participant or the pharmacist providing a summary of the conversation. See Additional file 1: Appendix A for the brief intervention protocol.

All trial pharmacists will be trained over half a day in the intervention protocol including flexible use of these discussion topics in ways influenced by the counselling approach of Motivational Interviewing [27]. A two hour evening follow-up session will also be scheduled shortly after the start of the trial. The purpose of the BI is to encourage participants to think further about their drinking and whether they should reduce it, and if they are ready to do so, to discuss how. It should be emphasised here that the pharmacists will not be trained in Motivational Interviewing, for which learning of skills cannot take place in such brief training workshops alone [28] This group will also be given the “Units and You” booklet [25], a “Unit/Calorie Calculator Wheel” [26] and an alcohol services leaflet to take away.

#### Control condition

Participants allocated to the control condition will not be explicitly informed that they are control participants. They will be given a leaflet entitled “Alcohol: The Basics” which includes information about alcohol. The leaflet content is *not* expected to be effective at promoting behaviour change [29].

### Outcomes

#### Primary outcomes

● Change in AUDIT scores between trial recruitment and follow-up.

● Proportion of hazardous and harmful drinkers (scoring 8 or higher on AUDIT) at follow-up.

#### Secondary outcomes

● AUDIT consumption subscale score.

● AUDIT problems subscale score.

● AUDIT dependence subscale score.

● General health status assessed using EQ-5D [30].

#### Data collection

Additional trial web pages have been developed and incorporated into the existing online system for pharmacists to record trial data [31]. The research team will have access to trial data by using secure usernames and passwords created for the trial. This online system has several advantages over the paper-pen method. RD will have access to pharmacy participants’ details as soon as each participant is randomised and the visit to the pharmacy completed; this will allow follow-up telephone calls to be made promptly and avoid delays caused by physically collecting completed trial paperwork from pharmacies. Access to randomisation status and baseline data is prevented. The system requires pharmacists to complete all fields correctly before proceeding to the next step, ensuring trial data are complete. Data can also be accessed without revealing randomisation status.

RD will arrange two telephone calls with study participants. During both calls the researcher will be blind to the participants’ intervention condition. The first telephone call will be made within two weeks of recruitment and will take between two to three minutes to complete. The purpose of this call will be to make personal contact to confirm contact details, gather additional tracing information as necessary and to arrange the follow-up study telephone call three-months post randomisation. Structured interview schedules will be used to ensure all participants are asked the same questions in a consistent manner in both calls. The primary and secondary outcomes specified above will be collected in the follow-up study telephone call after three months. This will take approximately 15 minutes to complete and participants will receive £10 gift voucher as a token of appreciation for completing the second call.

#### Randomisation

**Sequence generation** We expect each pharmacist to recruit 24 participants on average over a period of approximately 6 months, with equivalent numbers of intervention (*N* = 12) and control (*N* = 12) participants randomised for each pharmacist. The random sequence was generated in blocks of 12 (BI = 6, Control = 6) with an Excel program, with further blocks of the same size generated for those who recruited more participants.

**Allocation concealment mechanism** Only after the pharmacist has opened a sealed numbered envelope will randomisation status be revealed. Neither pharmacists nor pharmacy support staff will be able to subvert randomisation and choose which participant will receive BI if this process is implemented as designed. This will be monitored during pharmacy visits, when RD will check the sealed envelopes for evidence of tampering and that the envelopes have been opened in numerical order, and also by checking the integrity of the blocks via the online data entry system. In addition, pharmacists will receive training on the importance of following trial procedures.

#### Blinding

Pharmacists will be blind to allocation status until the point at which they open the sealed envelope. Thereafter they are not involved in research data collection. For the purposes of both follow-up study and data management relevant personnel will be blinded to randomisation status throughout the trial.

#### Statistical methods

The data will be analysed on an intention to treat basis. For outcome measures t-tests or Mann–Whitney tests will be used for continuous data and chi-square tests used for categorical data. If possible confounding is identified, ANCOVA and logistic regression will be used to examine the effect of the interventions over and above these other factors, and effect sizes and odds ratios calculated. For all tests, if necessary, measures will be transformed before analysis. All analyses not conforming to an *a priori* statistical plan will be declared as exploratory.

#### Sub-study

Participants who have consented to participate in a sub-study, on their experience of participating in the trial, will be randomly selected to be telephoned (*N* = 24). Consent for this will be obtained alongside consent to participate in the trial, i.e. in the consultation room with the pharmacist. They will be contacted approximately one month after the three-month follow-up telephone call by RD for a 20 minute discussion by telephone. A semi-structured topic guide developed for the study will be used to explore participants’ experience. The telephone interviews will be recorded using a digital voice recorder, transcribed verbatim, data managed using a qualitative research program [32] and analysed. Those who have completed the telephone interview will be sent a £10 gift voucher as a token of appreciation for participation.

#### Ethical and research governance approval

This study has been given a favourable ethical opinion (on 12^th^ December 2011, REC reference: 11/LO/1448) by the NRES Committee London- Queen Square, UK. NHS Permission was granted by The West London Primary Care Consortium (for Research and Innovation) on behalf of NHS Hammersmith and Fulham (Primary Care Trust) on 19^th^ March 2012, NIHR CSP number: 11839.

## Discussion

The UK Department of Health’s stated aim is to involve community pharmacists in the delivery of BI to reduce alcohol harms [3]. This will be the first RCT study to assess the effectiveness of BI delivered by community pharmacists and given this policy context, it is pragmatic in design. This setting attracts hazardous and harmful drinkers who are accessing community pharmacy services. There are minimal exclusion criteria, for example by not excluding those with co-morbidities, so the study population is similar as possible to those BIs would be delivered to if found to be effective.

The time available for pharmacists’ training (a morning on trial procedures and an afternoon on BI delivery with additional provision for a two hour evening follow-up) was negotiated mindful of time pressures and other commitments. Pharmacists will be reimbursed for their one day of training, and given a modest fee given for each trial participant. Similarly the duration of the BI has been tailored to the time available for interventions delivery in community pharmacies.

One possible consequence of these study design decisions is that a null finding may result because the effect of BI has been diluted by these study characteristics. We do not anticipate that setting-specific factors will interfere with intervention conduct, and the capacity of pharmacists to deliver BIs effectively is what we are studying. Although the primary care evidence-base is well established, there are many important questions about BI effectiveness that remain to be addressed [33,34]. An evaluation of a stronger intervention or with more extended training and skills development inputs may show greater effectiveness and an efficacy trial may show more promise. We are aware of these possibilities and have made our study design decisions as we judge most appropriate in the policy context described. If the trial produces evidence of effectiveness then it will be appropriate to subsequently undertake a large multi-centre trial evaluating the effectiveness and cost-effectiveness of this BI model to inform decision-making about the delivery of BIs in pharmacies nationally.

## Abbreviations

ANCOVA: Analysis of covariance;AUDIT: Alcohol Use Disorder Identification Test;BI: Brief Intervention;CLRN: Comprehensive Local Research Network;NHS: National Health Service;NIHR CSP: National Institute of Health Research Co-ordinated System for obtaining National Health Service Permission;NRES: National Research Ethics Services;REC: Research Ethics Committee;SASQ: Single Alcohol Screening Question;UK: United Kingdom;UKCRN: United Kingdom Central Research Network;W.H.O: World Health Organisation

## Competing interests

The authors declare that they have no competing interests.

## Authors’ contributions

RD, JM, IJN and CW designed the study. RD is the Chief Investigator for this study. RD and JM designed the intervention and delivered training to study pharmacists. RD and CW delivered training to pharmacy support staff. RD managed the trial including all liaison work with pharmacy staff at all sites. RD conducted all telephone follow-up interviews with study participants. RD wrote the first draft of this paper, JM revised the first draft and all authors contributed to successive drafts. All authors read and approved the final manuscript.

## Authors’ information

1. Ranjita Dhital, MSc, BSc (Pharmacy) MRPharmS (UK), Pharmacist and PhD student, King’s College London, Florence Nightingale School of Nursing and Midwifery, UK.

2. Ian. Norman, PhD, RN (UK), CQSW, FEANS, FRCN, FAAN, Professor, Deputy Head of School, King’s College London, Florence Nightingale School of Nursing and Midwifery, UK.

3. Cate Whittlesea, PhD, MSc Econ, BSc (Pharmacy) MRPharmS (UK), Senior Lecturer Pharmacy Practice, King’s College London, King’s Health Partners, Pharmaceutical Science Clinical Academic Group, Institute of Pharmaceutical Science, UK.

4. Jim McCambridge, PhD, MSc, PGDSW, PGCILT, BA, Senior Lecturer in Behaviour Change, London School of Hygiene & Tropical Medicine, Department of Social and Environmental Health Research, UK.

## Pre-publication history

The pre-publication history for this paper can be accessed here:

http://www.biomedcentral.com/1471-2458/13/152/prepub

## Supplementary Material

Additional file 1 Appendix A. Click here for file
