# Supplementary material for: Effectiveness of alcohol brief intervention delivered by community pharmacists: study protocol of a two-arm randomised controlled trial
Source: BMC Public Health. 2013 Feb 18;13:152. doi: 10.1186/1471-2458-13-152 (PMC3583737; doi:10.1186/1471-2458-13-152)
Supplement: Additional file 1 — Appendix A. [file 1471-2458-13-152-S1.docx]

**APPENDIX A**

**Brief Intervention Protocol**

**RECORD START TIME:……………….**

**GETTING STARTED**

1. **Start by building rapport and encourage informal chat. Your goal is to try help the person feel comfortable before talking to you about their drinking.**
2. **Identify customers’ experience of answering the AUDIT questions.**

Questions you **may** ask:

- ‘What was it like to answer these questions?’
- ‘Was there anything that made you think?’
- ‘Was there anything that struck you?’
- ‘What did you like or not like about answering these questions?’

Focus on questions with high scores:

- ‘For instance, your response to this question here?’

**CORE CONVERSATION**

1. **Encourage customer to talk about themselves:**

- ‘Why don’t we start by telling me a little bit about yourself?’
- ‘Where does your drinking fit in with this?’
- ‘Is it okay to talk a bit more about your drinking?’
- ‘Tell me a bit about what’s important in your life right now?’

1. **Explore ambivalence:**

- ‘What do you find positive about your drinking?’
- ‘What, do you find good about this?’
- ‘Tell me what’s negative about your drinking?’
- ‘What do you find not so good about that?’

1. **Evaluate their drinking:**

- ‘On balance what do you think about your drinking?’
- ‘How do you see your drinking now?’
- ‘What do you feel is important for you?’
- ‘What would be most helpful for you to talk about next?’

**CLOSING and SUMMARY**

1. **Ending the conversation:**
   - - - ‘Where do you feel you are right now with this?’
       - ‘Where does this leave you?’
       - ‘How did you find this?’
       - ‘How would you summarise this conversation?’

**Note**: If necessary, provide a summary to the customer of your understanding of their situation:

- ‘To make sure I’ve got it right…?’
- ‘I remember what you told me…’

**NOW COMPLETE THE BRIEF FORM ABOUT THIS CONVERSATION**

**RECORD END TIME: ……………… (try not to exceed 10 minutes)**

**Giving information and discussing printed literature**:

Restrict giving information and avoid going through the leaflets with the customer.

Provide information only in response to specific requests by the customer or if you judge it is likely to be helpful to them.

Draw from the following resources:

1. NHS Units and You Booklet
2. Unit/Calorie Calculator Wheel
3. Alcohol Services leaflet

Remember to seek permission first (e.g. ‘Is it okay to talk about X?’)

Questions you **may** ask:

- ‘Is there any information about alcohol you’d like to clarify?
- **‘**How might this information be useful to you?’ (before providing it)
- ‘What do you think about that?’ (after providing it)
- ‘Does that change how you think about your drinking at all?’

--------------------------------------------------------------------------------------------------------------------------

**Aims of the conversation**:

Aim to leave the customer thinking about their drinking and whether they would like to change their drinking in any way. If this has already been established, the more explicit their planning the better e.g. ‘I’m not going to drink anything this Friday night.’
